# Scar endometriosis

**DOI:** 10.4103/0970-9371.71877

**Published:** 2010-07

**Authors:** Zaheer Abbas Ali Khan Pathan, US Dinesh, Ravikala Rao

**Affiliations:** Department of Pathology, SDM College of Medical Sciences Dharwad, Karnataka, India

**Keywords:** Abdominal wall endometriosis, fine needle aspiration cytology, laparotomy scar

## Abstract

Endometriosis is the presence of functioning endometrium outside the uterus. Endometriosis rarely occurs in the abdominal wall. Majority of abdominal wall endometriosis occur in or adjacent to surgical scars, following caesarean section or hysterectomy. Laparotomy scar endometriosis following salpingectomy for ectopic pregnancy has rarely been reported. We report a case of scar endometriosis following laparotomy for chronic ectopic, and diagnosed by fine needle aspiration cytology (FNAC). Excision biopsy confirmed the FNAC diagnosis of scar endometriosis.

## Introduction

Endometriosis is defined as the presence of functioning endometrium outside the uterus.[1–4] It occurs in 8–15% of women of reproductive age group.[4–6] In spite of being relatively common, endometriosis remains a diagnostic and therapeutic enigma even today, largely due to its variable presentations.[4] Pelvis is the most common site.[3,4] Extrapelvic endometriosis is less common but more difficult to diagnose due to the extreme variability in presentation.[4] It is seen in lungs, bowel, ureter, brain and abdominal wall.[3] Endometriosis in an operative scar is rare.[2,5] Its clinical diagnosis is confused with abscess, hematoma, suture granuloma, desmoid tumor, sarcoma and metastatic malignancy.[3,7] We report a case of scar endometriosis in a woman who underwent laparotomy for chronic ectopic.

## Case Report

A 28-year-old (G2P1) female presented with a painful nodule of 2 years duration over the lower abdominal wall. She underwent laparotomy for chronic ectopic 3 years prior. Examination revealed a well-defined, 3×3 cm, firm and slightly tender nodule in the subcutaneous plane above the pfannenstiel scar. Ultrasonography revealed an ill-defined hypoechoic, 21×16 mm lesion in the subcutaneous plane. Patient was referred for fine needle aspiration cytology (FNAC) with a clinical diagnosis of desmoid tumor.

### Cytology

**Figure 1:**
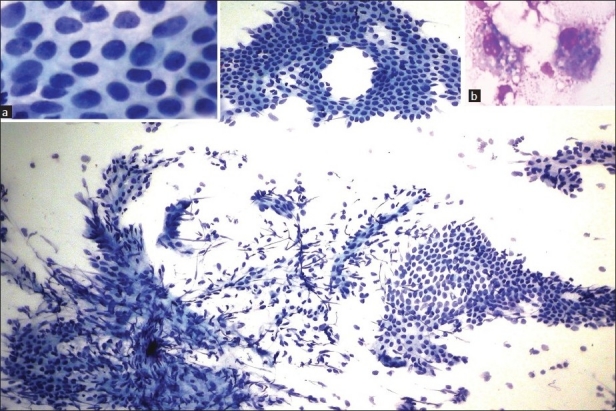
Microphotograph showing monolayered sheets of endometrial glandular cells and spindled stromal cells with capillary meshwork (Pap, ×100). Inset: (a) Epithelial cells (Pap, ×400) (b) hemosiderin laden macrophages (Leishman’s, ×400)

FNAC smears were cellular, showing monolayered sheets of polygonal epithelial cells having scant cytoplasm, uniform round to oval nuclei with inconspicuous nucleoli along with irregular stromal fragments of spindle cells with ovoid or elongated nuclei and moderate amount of cytoplasm arranged around prominent vascular network. Mixed inflammatory cell infiltrate, naked stromal nuclei and occasional hemosiderin laden macrophages were present in a hemorrhagic background [Figure 1]. Based on these features, FNAC diagnosis of endometriosis was offered.

### Histopathology

**Figure 2:**
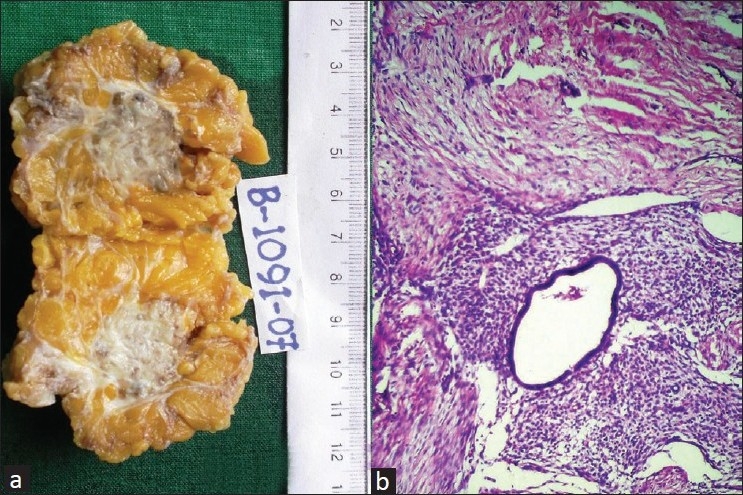
(a) Gross photograph showing grey-white fibrous area with tiny cysts in the subcutaneous fat (b) microphotograph showing endometrial glands with adjacent stroma in a fibrocollagenous background (H and E, ×100)

The excised mass was composed of 5×5 cm fibrofatty tissue with central fibrous grey-white area containing minute cystic spaces [Figure 2a]. Microscopy showed variably dilated endometrial glands surrounded by spindle cell stroma, lymphoplasmacytic infiltrate and hemosiderin laden macrophages within fibrocollagenous tissue [Figure 2b], confirming the FNAC diagnosis of endometriosis.

## Discussion

Extrapelvic endometriosis is an uncommon disorder with a prevalence of 8.9–15%.[1,7] It rarely involves bladder, kidney, omentum, bowel, lymph node, pleura, umbilicus, hernial sac and abdominal wall.[5,8] Endometriosis of the skin and soft tissue constitutes 3.5% cases of extrapelvic endometriosis.[6]

Endometriosis in a postoperative scar is rare. Majority of the reported cases have been observed in and adjacent to surgical scars following caesarean sections, hysterectomy, hysterotomy and rarely following surgeries on fallopian tube, appendicectomy, amniocentesis and episiotomy.[8]

The incidence of endometriosis developing in the scar depends on the indication for the original surgery, being 1.08% for mid-trimester abortion and 0.03–0.4% following caesarean sections. The higher incidence in mid-trimester abortions may be due to pluripotential capability of early deciduas, resulting in cellular replication producing endometriomas.[7,8]

The first case of scar endometriosis was reported by Meyer in 1903.[9] Blanco *et al*.[7] reported 10 cases of scar endometriosis of which 9 cases followed caesarean section and one occurred in laparotomy for ectopic pregnancy. In a study by Pathan *et al*.,[5] seven cases occurred in caesarean and one occurred in a hysterectomy scar. Horton *et al*.[3] reviewed 445 cases of abdominal wall endometriosis among which 57, 11 and 12% cases occurred in scars of caesarean section, hysterectomy and other surgical procedures, respectively. Twenty percent cases did not occur in the scar but elsewhere such as umbilicus and the groin.

Two theories concerning the pathogenesis have been proposed: (1) the most favored metastatic theory states the transport of endometrial cells to adjacent locations via surgical manipulations, hematogenous or lymphatic dissemination and (2) primitive pluripotential mesenchymal cells undergo specialised differentiation and metaplasia into endometrial tissue (metaplastic theory).[6]

Clinically, the features diagnostic of scar endometriosis are lump in the scar, pain, increasing size of lump, bleeding and skin discoloration. Cyclicity of symptoms during menstruation is not characteristically seen in all cases, however, if present, is pathognomonic of scar endometriosis.[1] The interval between onset of symptoms and patient’s index surgery varies from 3 months to 10 years.[8] Clinically, the lesion appears as a firm nodule and hence can be easily evaluated by FNAC.[7] This will help in differentiating it from metastatic disease, desmoid tumor, lipoma, sarcoma, cysts, nodular and proliferative fasciitis, fat necrosis, hematoma or abscess.[5,6]

Smears from the endometriomas show varying cellularity comprising epithelial and spindle stromal cells, with variable number of hemosiderin laden macrophages and inflammatory cells.[6,10] The presence of any two of the three components (endometrial glands, stromal cells and hemosiderin laden macrophages) has been used for the cytological diagnosis of endometriosis.[5] The cytological features of scar endometriosis are related to cyclical hormonal changes. In proliferative phase, the epithelial cells form cohesive sheets of uniform small cells with scant cytoplasm, round to ovoid nuclei with bland chromatin and occasional non-atypical mitosis. During secretory phase, the cell size gradually increases with cytoplasmic microvacuolations. The stromal cells show abundant cytoplasm and predecidual change with an epithelioid appearance, causing diagnostic difficulties. The background is generally sanguineous, contains inflammatory cells and histiocytes (with/or without hemosiderin). Squamous, tubal and mucinous metaplasia and isolated cases of malignant transformation in scar endometriosis have been reported.[6]

The lesions in the differential diagnosis of mass associated with abdominal scar have well-defined cytological features. Desmoid tumor and fibrosis show less cellularity with benign appearing mesenchymal cells. Suture granuloma shows nonspecific inflammation with or without granulomatous elements and foreign material. Fat necrosis shows foamy macrophages, inflammatory and multinucleate giant cells, fragments of adipose tissue and no epithelial cells. Nodular fasciitis shows myxoid background and pleomorphic cells. Smears from primary or metastatic malignancies show hypercellularity with frankly neoplastic cells.[6]

The imaging modalities are non-specific but useful in determining the extent of the disease and planning of operative resection, especially in recurrent and large lesions.[7] So, FNAC may be the only diagnostic tool in the evaluation of these lesions, providing rapid and accurate preoperative diagnosis.

The treatment of choice is wide local excision.[6,7] Abdominal wall musculature involvement requires *en bloc* resection of the myofascial elements.[3]

## Conclusions

Scar endometriosis is an uncommon condition that primarily affects women of reproductive age. Patients usually present 2–5 years following uterine or fallopian tube surgery, with a painful nodule that may become more symptomatic during menstruation. FNAC is a relatively inexpensive, less traumatic, rapid and accurate diagnostic tool for diagnosis and to rule out other common conditions.
